# Integrated design-sense-plan architecture for autonomous geometric-semantic mapping with UAVs

**DOI:** 10.3389/frobt.2022.911974

**Published:** 2022-09-08

**Authors:** Rui Pimentel de Figueiredo, Jonas Le Fevre Sejersen, Jakob Grimm Hansen, Martim Brandão

**Affiliations:** ^1^ Department of Electrical and Computer Engineering, Aarhus University, Aarhus, Denmark; ^2^ Department of Informatics, King’s College London, London, United Kingdom

**Keywords:** next-best-view planning, Autonomous Aerial Vehicles (AAV), design-optimization, deep learning, semantic volumetric representation, multi-camera systems

## Abstract

This article presents a complete solution for autonomous mapping and inspection tasks, namely a lightweight multi-camera drone design coupled with computationally efficient planning algorithms and environment representations for enhanced autonomous navigation in exploration and mapping tasks. The proposed system utilizes state-of-the-art Next-Best-View (NBV) planning techniques, with geometric and semantic segmentation information computed with Deep Convolutional Neural Networks (DCNNs) to improve the environment map representation. The main contributions of this article are the following. First, we propose a novel efficient sensor observation model and a utility function that encodes the expected information gains from observations taken from specific viewpoints. Second, we propose a reward function that incorporates both geometric and semantic probabilistic information provided by a DCNN for semantic segmentation that operates in close to real-time. The incorporation of semantics in the environment representation enables biasing exploration towards specific object categories while disregarding task-irrelevant ones during path planning. Experiments in both a virtual and a real scenario demonstrate the benefits on reconstruction accuracy of using semantics for biasing exploration towards task-relevant objects, when compared with purely geometric state-of-the-art methods. Finally, we present a unified approach for the selection of the number of cameras on a UAV, to optimize the balance between power consumption, flight-time duration, and exploration and mapping performance trade-offs. Unlike previous design optimization approaches, our method is couples with the sense and plan algorithms. The proposed system and general formulations can be be applied in the mapping, exploration, and inspection of any type of environment, as long as environment dependent semantic training data are available, with demonstrated successful applicability in the inspection of dry dock shipyard environments.

## 1 Introduction

UAVs deployed in natural, industrial and urban contexts are challenged with increasingly complex scenarios and tasks. Deciding which regions of the environment to cover during visual search and mapping tasks is computationally demanding. Therefore, UAVs should be equipped with efficient active exploration mechanisms that for improved visual search for objects of interest while building detailed semantic maps of the scene and avoiding the potential computational overload of processing irrelevant sensory information. They also should be equipped with an appropriate number of sensors that take into account increased mapping performance and battery constraints. This article is focused on multi-camera drone design, as well as on developing autonomous navigation and environment representations for inspection applications focused in shipyard scenarios using flying multi-camera systems. The use of autonomous UAVs in inspection tasks can improve efficiency and task-execution speed without compromising quality. This work addresses the implementation and evaluation of the viability of multi-camera autonomous platform for inspection tasks.

**FIGURE 1:**
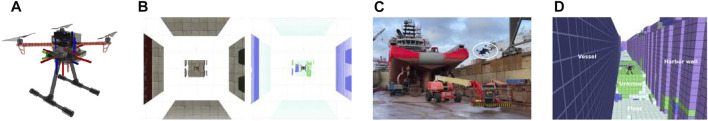
A UAV platform endowed with a multi stereo camera system for autonomous drone navigation applications. **(A)** The proposed design is general enough to easily attaching a total of 5 stereo vision cameras around the UAV frame (i.e., front, left, right, back, bottom) for **(B)** high field-of-view RGB-D and semantic visual analysis. **(C)** Live-mission image of an autonomous UAV dockyard inspection task using our **(D)** semantic-aware receding horizon path planner and volumetric-semantic mapping representation.

High visual coverage for improved mapping and navigation can be obtained using a single drone with multiple cameras at the cost of augmented payload and additional computation power. In this work, we propose a systematic solution for autonomous ship inspection tasks using a single multi-camera UAV, that uses state-of-the-art simultaneous localization and mapping (SLAM) techniques with cost-efficient NBV exploration algorithms to efficiently label and geometrically reconstruct all objects in shipyard environments using a novel utility function that balances exploration and exploitation, using both geometric and semantic probabilistic information (see Figure 1). Semantic information is useful to bias exploration towards specific known object types.

We propose a full integrated approach for autonomous exploration and mapping that considers design, sensing, and path planning of multi-camera UAV system, in a coupled manner. Our mapping system is targeted at multi-camera UAVs, includes probabilistic semantic-metric mapping representations, and uses a path planning algorithm that considers both semantic and geometric information for autonomous environment reconstruction. Our target application is the inspection of vessel structures in shipyard environments, requiring minimal human intervention.

Our method relies on an efficient probabilistic observation model for depth and semantic information that allows fast computation of probabilistic measurements and continuous Bayesian fusion on probabilistic metric-semantic grid mapping structures,A environment representation that facilitates Bayesian fusion, in memory-efficient 3D encoding volumetric and semantic information provided by consumer-grade RGB-D sensors and state-of-the-art DCNNs for 2D scene semantic segmentation. A real-time, path planning approach that considers both geometric and semantic probabilistic information, in a receding-horizon probabilistic fashion, using rapid random trees (RRTs). Our flexible method allows biasing exploration towards specifically known object classes, while avoiding task-irrelevant ones. Our main contributions with respect to our previous work ? are:

1. An extensive evaluation on the trade-offs of using multi-camera UAVs, from a power consumption, computational and exploration and mapping performance perspective.
2. Design decisions for the selection of the number of cameras on a UAV, based on power consumption, flight-time duration, and exploration and mapping performance constraints and trade-offs. To our knowledge this study is of the utmost importance for resource constrained unmanned applications, and this work is the first assessing the former problem trade-offs on an UAV application scenario.

Our probabilistic observation model approximates the 3D covariance matrix by its principle component, corresponding to the predominant axial noise. This allows fast probabilistic fusion of 1-dimensional noise in 3D grids. Unlike previous RRT based planning methods, our hybrid approach accounts for both semantic and geometric data, using information theoretical principles, and prioritizes attention to high entropy regions. The reminder of this article is structured in the following manner: first, in Section 2, we revise state-of-the-art works on semantic and geometric environment representations as well as NBV planning algorithms for autonomous mapping and robotics applications. Then, in Section 3 we describe in detail the proposed methodologies for autonomous exploration. The results in Section 4 evaluates the solutions for autonomous mapping and navigation, in a realistic simulated shipyard environment. Finally, discuss our main contributions, limitations of our methods, and future improvements in the final conclusions section.

## 2 Related work

Environment representations and active vision methodologies for autonomous exploration and mapping used in navigation applications are thoroughly evaluated in the remainder of this section.

### 2.1 Drone applications

Today, there exist numerous of different applications for aerial drones. Among those are the maritime industry Chamoso et al. (2014), agriculture Mogili and Deepak (2018), traffic surveillance Bozcan and Kayacan (2020), and construction management Li and Liu (2019).

Collaborative operation of a set of UAVs was proposed for detecting oil spills in the sea Chamoso et al. (2014). Within precision agriculture problems, crop monitoring, crop height estimations, and pesticide spraying are some of the most important tasks. In the work Mogili and Deepak (2018) the authors state the various important components embedded in the UAV, such as IMUs for measuring angular rates and forces, a magnetometer to measure the magnetic field, GPS to provide geo-location, camera to capture motion and objects, 2D Laser scanners to capture the shape of objects, and barometer to measure pressure.

In construction management logistics, on-site constructions, maintenance, and demolition are investigating in order to discover the potential optimization in how UAVs can achieve cost-effective solutions and to cut carbon emissions Li and Liu (2019). 3D models can be created from 2D imaging data by aerial photogrammetry. Having a generic solution across applications is of the utmost importance since there are many different applications tasks to be handled. However, when diving into different applications, specific accuracy requirements require customized solutions.

The low-level control of the UAVs is typically not in focus when dealing with high-level autonomy in industrial applications. A micro-controller based off-the-shelf solution is normally chosen. High-level goals, such as way-points, have the maximum latency budget and are sent to the low-level flight controller. For instance, PX4 Meier et al. (2015) is a low-cost standardized platform supporting ROS Quigley (2009), which is a node-based multi-threaded open-source robotics framework for deeply embedded platforms, adopted in this work.

### 2.2 Mapping representations

Visual mapping representations are essential long-term memory mechanisms in robotics navigation tasks such as inspection, as we target in this article.

#### 2.2.1 Metric representations

The most used metric mapping representation in the literature is called probabilistic 3D occupancy grids. These, represent the environment as cells, each one having a binary state representing occupied and free space, being popular for autonomous navigation since access, memory use, collision checking, and path planning can be made efficient through the use of octree data structures Hornung et al. (2013).

Elevation maps Herbert et al. (1989) are a more compact 2.5D representation that encodes probabilistic height-levels on a 2D grid Michel et al. (2005), being convenient for legged locomotion applications Gutmann et al. (2005). Nevertheless, these are typically unsuitable for applications where the agent has to navigate between objects at distinct heights (e.g., complex infrastructures or natural environments). Multi-level surface maps Triebel et al. (2006) overcome this setback by relying on a list of heights for each cell. Despite being cheap in terms of memory use, their main drawback resides on the impossibility of explicitly distinguishing between unknown and free space, which is essential for environment exploration and safe navigation tasks. Recently, the idea of using continuous representations in mapping has also attracted great attention in the robotics community O’Callaghan et al. (2009).

#### 2.2.2 Semantic representations

The methods for semantic segmentation existing in the literature can be divided as follows. Methods based on 2D grayscale or RGB images and methods based on RGB-D point cloud data.

State-of-the-art methods for image segmentation are based on deep-learning architectures Ronneberger et al. (2015); He et al. (2017) that learn from large annotated datasets to regress from 2D images to object masks that encode the layout of input objects. Mask R-CNN He et al. (2017), for instance, extracts a set of blobs from images, each associated with a class label, a bounding box, and the object mask (i.e., segmentation). It combines ResNet He et al. (2016) or a Region Proposal Network (RPN) Ren et al. (2017) backbone for feature extraction (which is shared across the three stages) with a Fully Convolutional Networks Long et al. (2015) for semantic segmentation, which uses a per-pixel softmax and multinomial cross-entropy losses.

Semantic-metric representations attempt to combine both geometric with semantically meaningful information Ashour et al. (2020). Volumetric Convolutional Neural Networks (CNNs) for 3D object classification and segmentation Maturana and Scherer (2015); Zhirong et al. (2015); Charles et al. (2017) are based on the idea of using CNNs on voxelized structures. However, these representations are constrained by their resolution and computational cost of expensive 3D convolutions, being currently unsuitable for real-time applications. In this work, we use BiseNet Yu et al. (2018) for image semantic-segmentation since it is robust, fast and small in size Hu et al. (2019), and easy usage, making it suitable for perception applications running on embedded systems (e.g., UAVs) with low budget computational specifications. To obtain a 3D representation in real-time, we fuse the 2D semantic information provided by BiseNet with depth measurements provided by consumer-grade RGB-D cameras, which are efficiently fused in a probabilistic 3D octogrid structure Hornung et al. (2013). Supervised training of deep neural networks rely on the availability of large annotated data sets, hand-labeled in a laborious and time consuming manner, which may be impracticable for applications and machine learning tools, requiring large data sets. Therefore, we explore the use of synthetic data, generated in a realistic virtual environment to overcome the reality gap.

### 2.3 Active perception

In this article, we tackle the problem of controlling the viewpoint(s) of a sensor(s) to improve task performance. The active vision problem is of the utmost importance in robotics applications Scott et al. (2003); Chen et al. (2011), and has been continuously redefined since the work of Aloimonos et al. (1988). More specifically, our goal is to autonomously decide where to next move a UAV at each point in time to improve task performance, according to some criteria—in our case, geometric and semantic mapping quality.

#### 2.3.1 Next-best-view planning

NBV planning has been widely studied by the robotics community and plays a role of primordial importance on object reconstruction Hou et al. (2019), autonomous mapping Isler et al. (2016), and safe navigation Brandão et al. (2020) tasks, to name a few.

Existing NBV approaches Delmerico et al. (2018) may be categorized as one of the following: Frontier and information-driven planning. Frontier-based planners Yamauchi (1997); Dornhege and Kleiner (2013) guide the cognitive agent to frontiers between unknown and free space, which benefits exploration. Information-driven methods rely on probabilistic environment representations and select the views that maximize expected information gains Delmerico et al. (2018) by using ray casting to back-project probabilistic volumetric information on candidate views. However, most existing approaches differ in the information gain definition. One way of tackling the problem is to incrementally compute and target a sensor at the next best view according to some criteria (e.g., optimize reconstruction quality). For example, Brandao et al. (2013) proposes a NBV algorithm that greedily targets the gaze of a humanoid robot at points of maximum entropy along a path. Other work proposes to use the average information-theoretic entropy over all voxels traversed via ray casting Kriegel et al. (2015). Such an approach has been further extended to include the incorporation of visibility probabilities as well as probability of seeing new parts of the object Isler et al. (2016). Recent work also extends information gain with considerations of self-occlusion and applies it to sensor scheduling Brandão et al. (2020).

Incremental techniques such as random tree sampling LaValle (1998) build tree representations of space using sampling. Such methods include Rapidly-exploring Random Tree (RRT) methods, or their variants RRT* Karaman and Frazzoli (2011) and RRT-Connect Kuffner and LaValle (2000). Since considering all the possible views is computationally intractable, RRT-based methods consider a subset of all possible views at each planning step. The tree is randomly expanded throughout the exploration space. In NBV methods, a utility function is used during the expansion of the tree to compute the expected information gain of sensor views according to some criteria.

In the works closest to ours, of Bircher et al. (2018), the authors propose a receding horizon “next-best-view” planner for UAVs that computes a random tree in an online real-time manner. The quality of each node in the tree is determined by the amount of unmapped space captured by the path the sensor makes until that location. A receding-horizon approach is then used—at each planning step, the first edge of the best branch is executed until the complete exploration of the environment is achieved.

## 3 Methodologies

In the rest of this section, we describe the proposed system and methodologies for active exploration and semantic-metric mapping.

### 3.1 System overview

**FIGURE 2:**
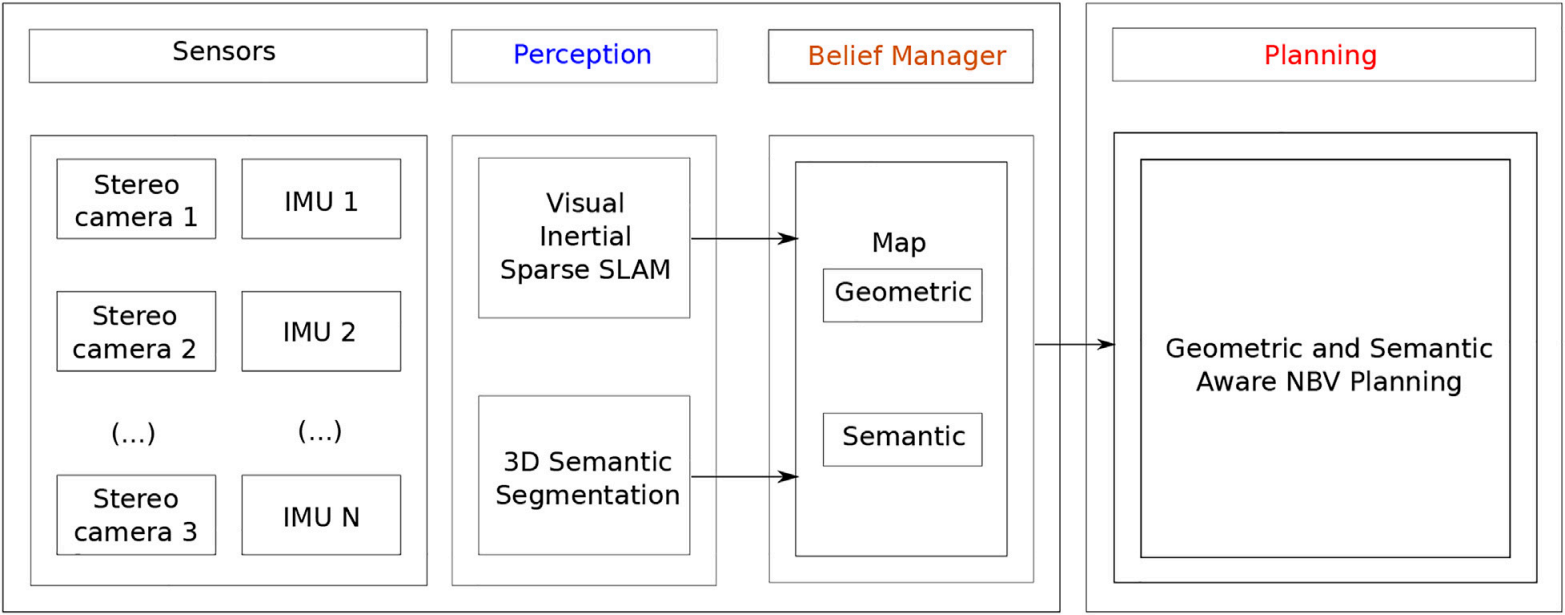
Overview of the proposed autonomous navigation system for localization and semantic-metric mapping of shipyard environments.

The proposed drone system for autonomous inspection is depicted in Figure 2, and consists of a UAV built for mapping tasks, that comprises multiple cameras, Inertial Motion Units (IMUs), and an altimeter (see Figure 9). Our navigation system relies on an off-the-shelf visual-inertial SLAM system with loop closing, and re-localization capabilities Mur-Artal and Tardós (2017), which is fed with RGB-D data provided by multiple stereo cameras and IMUs measurements, for improved robustness on self-motion tracking performance Bloesch et al. (2015).

Our probabilistic observation model that combines metric and semantic visual cues, which are efficiently fused in a volumetric octogrid structure Hornung et al. (2013), and a NBV planner that uses both geometric and semantics for exploration.

### 3.2 Multi-camera drone system design

In our CAD model, we attempt to minimize the weight to achieve better flight performance and flight duration. At the same time, we want to minimize the length of parts to avoid vibrations while ensuring we can mount camera sensors in the location and orientation we want. Camera sensors are attached to the quad base frame (DJI F450), together with a battery mount for easy battery change. We use Jetson Xavier NX as our onboard computer and the Pixhawk 4 as the low-level flight controller (see Figure 9).

The system utilizes a set of camera sensors

$$S=\left\{S^{1},\ldots ,S^{N_{s}}\right\}$$ (1)

which are rigidly attached to the UAV body base frame B , and whose poses are assumed deterministic and known from the kinematics model.

### 3.3 Probabilistic volumetric-semantic occupancy mapping

To represent the environment enclosing the autonomous planning agent we consider a 3D uniform voxel grid structure. Let *m* = {*m*_*i*_} be the environment map data structure, where each voxel $m_{i}=\left\{m_{i}^{o},m_{i}^{s}\right\}$ with $m_{i}^{o}\in \left\{0,1\right\}$ denoting a random occupancy variable, and $m_{i}^{s}\in \left\{1,\ldots ,K_{c}\right\}$ a semantic variable representing object class. Recursive Bayesian volumetric mapping Thrun et al. (2005) is employed to sequentially compute the posterior probability distribution over the map, given sensor observations $z_{1:t}=\left\{z_{1:t}^{o,1},\ldots ,z_{1:t}^{o,N_{s}};z_{1:t}^{s,1},\ldots ,z_{1:t}^{s,N_{s}}\right\}$ and sensor poses $p_{1:t}=\left\{p_{1:t}^{1},\ldots ,p_{1:t}^{N_{s}}\right\}$ obtained through the robot kinematics model and an off-the-shelf SLAM module, from time 1 to *t*

$$P\left(m|z_{1:t},p_{1:t}\right)=\underset{i}{\prod }P\left(m_{i}|z_{1:t},p_{1:t}\right)$$ (2)

considering the occupancy of cells are independent. Updates can be recursively computed in log-odds space Moravec and Elfes (1985) to ensure numerical stability and efficiency using the following iterative probabilistic sensor fusion model

$$L\left(m_{i}|z_{1:t},p_{1:t}\right)=L\left(m_{i}|z_{1:t-1},p_{1:t-1}\right)+L\left(m_{i}|z_{t},p_{t}\right)+L\left(m_{i}\right)$$ (3)

with

$$L\left(.\right)=\text{log}\left[\frac{P\left(.\right)}{1-P\left(.\right)}\right]$$

where *L* (*m*_*i*_|*z*_*t*_, *p*_*t*_) represents the inverse sensor model, *L* (*m*_*i*_|*z*_1:*t*−1_, *p*_1:*t*−1_) represents the recursive term and *L* (*m*_*i*_) the prior. Considering that the map is initially unknown, i.e. *P*(*m*_*i*_) = 0.5, eliminates the last term of Eq. 3.

### 3.4 3D semantic segmentation

Our method for semantic segmentation relies on a DCNN encoder-decoder segmentation network, named BiseNet Yu et al. (2018), that receives RGB or grayscale images as input, and first encodes the image information and then decodes it again, and outputs a probability distribution over the known object categories for each pixel (*u*, *v*).

BiseNet comprises two different branches. The spatial pathway which encodes low-level information, and the context one which mainly encodes high-level context information. A Feature Fusion Module is used to fuse features from these two paths. First, these are concatenated, and then batch normalization is used to balance the scale of the features. Finally, the concatenated features are pooled and re-weighted using a weight vector.

In this work, we use BiseNet because it is compact, fast, robust, and easy to use, being suitable for remote sensing applications running on embedded systems (e.g., UAVs) with low computational specifications Hu et al. (2019). For each pixel (*u*, *v*), the network outputs a probability distribution $p^{c}\left(u,v\right)\in P^{K_{c}}$ over the set of known classes C , where *K*_*c*_ represents the number of known classes. For training the network, we use the categorical Cross-Entropy loss function

$$CE=-\text{log}\left(\frac{e^{s_{p}}}{\sum_{j}^{C}e^{s_{j}}}\right)$$ (4)

where *s*_*j*_ is the CNN output score for the class j∈C , and *s*_*p*_ the positive class.

**FIGURE 3:**
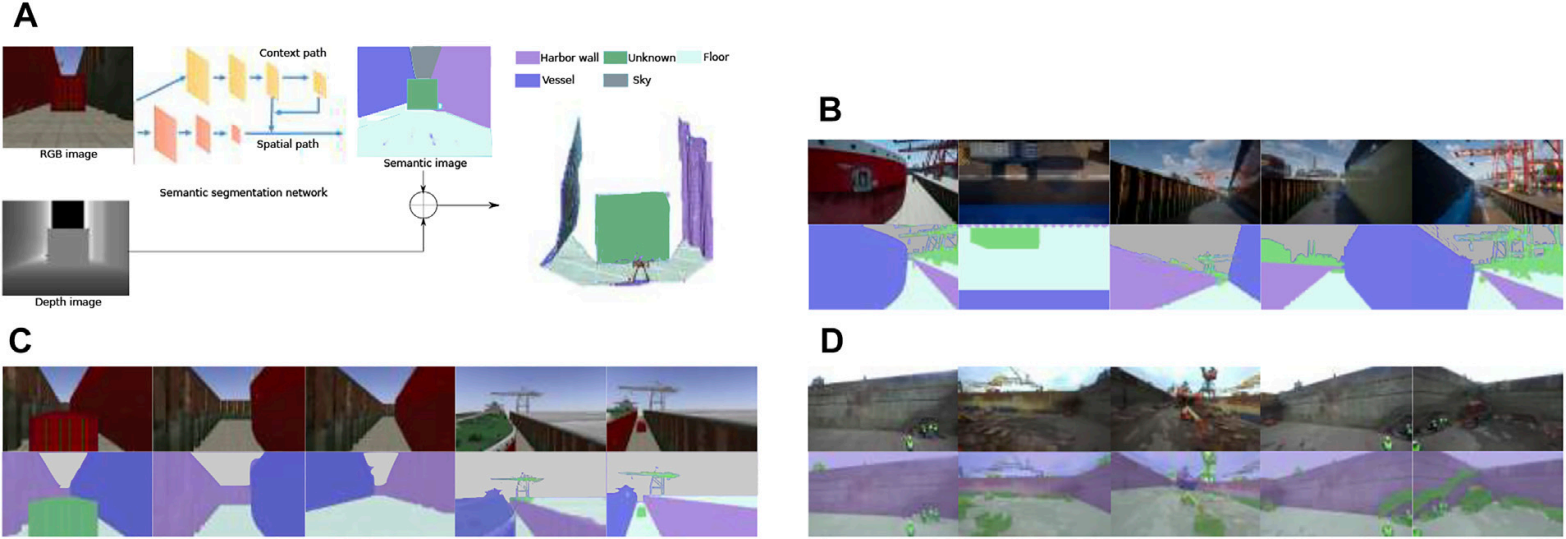
**(A)** Our architecture for 3D semantic segmentation. Sample training images collected in **(B)** Unreal Engine, **(C)** Gazebo, and a **(D)** real environment, respectively.

At run-time, the probability distributions over all classes and image pixels, gathered with BiseNet, are fused with the corresponding depth image, from known extrinsics, to obtain a semantic point cloud (see Figure 3A). Each resulting point cloud data point comprises geometric (3D), color (RGB), and semantic (multi-class) information.

### 3.5 Efficient probabilistic sensor fusion model

Our sensor depth noise model is based on the one proposed in Nguyen et al. (2012), which considers that single point measurements $z_{t,k}^{o}$ are normally, independent, and identically distributed (iid) according to

$$z_{t,k}^{o}|m,p_{t}∼N\left(z_{t,k}^{o\text{*}};\Sigma_{t,k}^{o}\right)$$ (5)

with

$$\Sigma_{t,k}^{o}=\text{diag}\left(\sigma_{t,k}^{l},\sigma_{t,k}^{l},\sigma_{t,k}^{a}\right)$$

where $z_{t,k}^{o\text{*}}$ denotes the true location of the measurement and $\sigma_{t,k}^{l}$ and $\sigma_{t,k}^{a}$ represent the lateral and axial noise standard deviations, respectively.

With the view of reducing computational complexity, we consider that noise is predominant in the axial direction (*σ* ^*a*^ ≫ *σ* ^*l*^), corresponding to the main component of the covariance matrix. This assumption allows approximating the 3D covariance matrix by a 1D variance and rely on cost efficient 1D cumulative distributions, for efficient probabilistic Gaussian fusion on 3D volumetric maps. For each measurement $z_{t,k}^{o}$ , we then update the corresponding closest grid cell *m*_*i*_ with the probability enclosed within the cell volume approximated as follows

$$P\left(m_{i}|z_{t,k}^{o},p_{t}\right)\approx F_{z}\left(z_{t,k}^{o}+\frac{\delta }{2}\right)-F_{z}\left(z_{t,k}^{o}-\frac{\delta }{2}\right)$$ (6)

where *δ* represents the grid resolution and *F*_*z*_ (.) the cumulative normal distribution function of *z*, where the axial error standard deviation can be approximated by a simple quadratic model

$$\sigma_{t,k}^{a}\approx \lambda_{a}\|z_{t,k}^{o}{\|}^{2}$$ (7)

with *λ*_*a*_ being a sensor specific scaling factor. All other cells belonging to the set of voxels traversed through ray casting (from the origin to endpoint $z_{i}^{o}$ ) are updated as being free with probability *P*_free_. This approximation considers that map resolution and perceptual noise have the same magnitude while at the same time allowing to reduce the computational burden of sensory fusion. The associated semantic measurements $z_{t,k}^{s}$ , with probabilities given by $P\left(z_{t,k}^{s}|I_{t},p_{t}\right)=\left\{P_{1},\ldots ,P_{K_{c}}\right\}$ are independently updated, according to

$$P\left(m_{i,t}^{s}|z_{t,k}^{s},p_{t}\right)=\eta P\left(z_{t,k}^{s}|I_{t},p_{t}\right)P\left(m_{i,t-1}^{s}|z_{1:t-1}^{s},p_{1:t-1}\right)$$ (8)

where *η* is a normalizing constant. The semantic probabilities can be updated efficiently in log-odds space $\left[L_{s_{0}},\ldots ,L_{s_{K}}\right]$ , for each class *k* and cell *i* according to:

$$\begin{array}{ll} L_{s_{k}}\left(m_{i,t}^{s,k}|z_{1:t}^{s,k},p_{t}\right) & =L_{s_{k}}\left(m_{i,t-1}^{s,k}|z_{t-1,k}^{s},p_{t-1}\right)+\\ + & L_{s_{k}}\left(m_{i,t}^{s,k}|z_{t,k}^{s},p_{t}\right)+L_{s_{k}}\left(m_{i,0}^{s,k}\right) \end{array}$$ (9)

where the inverse sensor model is given by

$$L_{s_{k}}\left(m_{i,t}^{s,k}|z_{t,k}^{s},p_{t}\right)=\text{log}\left(\frac{P\left(m_{i,t}^{s,k}|z_{t,k}^{s},p_{t}\right)}{P\left(m_{i,t}^{s,0}|z_{t,0}^{s},p_{t}\right)}\right)$$ (10)

and where *k* = 0 represents the pivot class (e.g., the unknown object class).

### 3.6 Semantic-Aware next-best-view planning

The proposed receding horizon NBV planner is based on Bircher et al. (2018). At each viewpoint, the planner generates a set of rays *R* that end if they collide against a physical surface or reach the limit of the map.

For a given occupancy map representing the world *m*, the set of visible and unmapped voxels from configuration *ξ*_*k*_ is Visible (*m*, *ξ*_*k*_). Every voxel *m*_*i*_ in this set lies in the unmapped exploration, and its ray does not cross occupied voxels. In addition, it complies with the sensor observation model (i.e., inside the Field of View (FOV) and maximum sensor range). The expected information gain Gain(*n*_*k*_) for a given tree node is the cumulative volumetric gain collected along the rays cast from *ξ*_*k*_, according to

$$\text{Gain}\left(n_{k}\right)=\text{Gain}\left(n_{k-1}\right)+G\left(\text{Visible}\left(m,\xi_{k}\right)\right)e^{-\lambda \text{cost}\left(\sigma_{k-1}^{k}\right)}$$ (11)

where *n*_*k*_ is defined as the node *k* in the RRT, and *G* (Visible (*m*, *ξ*_*k*_)) is the local information gain obtained from the visible and unmapped voxels in map *m* seen from configuration *ξ*_*k*_ (i.e., pose of the camera at node *n*_*k*_). The original formulation of Bircher et al. (2016) defines the local information gain as

$$G\left(\text{Visible}\left(m,\xi_{k}\right)\right)=\underset{i}{\sum }I_{g}\left(m_{i}\right)$$ (12)

with

$$I_{g}\left(m_{i}\right)=\left\{\begin{array}{cc} 1\;\; & \text{if }P\left(m_{i}^{o}\right)=0.5\left(\text{i.e. unknown}\right)\\ 0\;\; & \text{otherwise }\left(\text{i.e. free or occupied}\right) \end{array}\right.$$ (13)

Our approach main difference resides on the information gain definition. It leverages both the volumetric and semantic information entailed by each voxel. We model volumetric entropy at each voxel *m*_*i*_ as

$$H^{o}\left(m_{i}\right)=-P\left(m_{i}^{o}\right)ln\left(P\left(m_{i}^{o}\right)\right)$$ (14)

and the semantic entropy as a sum over per-class entropy

$$H^{s}\left(m_{i}\right)=\overset{K_{C}}{\underset{k=1}{\sum }}H_{k}^{s}\left(m_{i}\right)$$ (15)

with per class-entropy $H_{k}^{s}\left(m_{i}\right)$ equal to

$$H_{k}^{s}\left(m_{i}\right)=-P\left(m_{i}^{s_{k}}\right)ln\left(P\left(m_{i}^{s_{k}}\right)\right)$$ (16)

where $P\left(m_{i}^{s_{k}}\right)$ is the probability of cell *i* being of class *k*.

We propose two different probabilistic information gain formulations. The first alternative accounts only for the occupancy information the voxel provides, according to

$$I_{g}\left(m_{i}\right)=-H^{o}\left(m_{i}\right)$$ (17)

The second formulation incorporates semantic constraints as a weighted summation of the information gain per class, across all voxels:

$$I_{g}\left(m_{i}\right)=H^{o}\left(m_{i}\right)\underset{k}{\sum }w_{k}^{s}H_{k}^{s}\left(m_{i}\right)\;\underset{k}{\sum }w_{k}^{s}=1$$ (18)

where $w^{s}=\left[w_{1}^{s},\ldots ,w_{K_{c}}^{s}\right]$ corresponds to a user specified weight vector, representing task-dependent class specific exploration biases.

As in Bircher et al. (2018), the path that is selected by the UAV is picked by choosing the largest cumulative-gain node in the RRT and executing the first segment of the path towards that node in a receding-horizon manner.

Finally, compared to Bircher et al. (2018), instead of sampling position and orientation uniformly in the RRT, we generate positions uniformly but greedily pick the orientation that leads to the highest gain from a quantized set of pre-defined number orientations.

## 4 Experiments

In this section, we perform a set of experiments in a realistic virtual environment to assess the performance of the proposed approaches in a UAV-based dockyard inspection scenario. All experiments were run on an Intel^®^ i7-10875H CPU with a GeForce RTX 2080 graphics card.

### 4.1 Semantic segmentation

#### 4.1.1 Semantic segmentation evaluation metrics

In order to access the performance of the image segmentation module, we rely on the pixel accuracy *P*_acc_(*C*) metric:

$$P_{\text{acc}}\left(c\right)=\frac{\#\text{TP}\left(c\right)+\#\text{TN}\left(c\right)}{\#\text{TP}\left(c\right)+\#\text{TN}\left(c\right)+\#\text{FP}\left(c\right)+\#\text{FN}\left(c\right)}$$ (19)

where true positive (TP), false positive (FP), true negative (TN), and false negatives (FN) represent pixels classified correctly as *c*, incorrectly as *c*, correctly as not *c*, and incorrectly as not *c*, respectively.

### 4.1.2 Semantic datasets description

The dataset utilized for training our semantic segmentation network is generated using a combination of Unreal Engine 4 Gestwicki (2019) and AirSim Shah et al. (2018) to create a realistic virtual dockyard environment, and to extract images, and the corresponding labels. Figures 3B–D shows examples of generated images. On the top is the image captured from the environment, and on the bottom the corresponding labeled image.

The UAV used for recording the dataset contains five cameras, four mounted on each side of the drone and one on the bottom. These cameras generate images at 1Hz while the UAV is performing the inspection route from different heights. The environment consists of a shipyard comprising different vessels with different textures, containers, cranes, and tiled/concrete floor, as well as multiple objects (e.g., puddles of water) scattered around on the floor. To increase the dataset variability, shadows and illumination conditions are dynamically changed based on the simulated daytime and weather.

**FIGURE 4:**
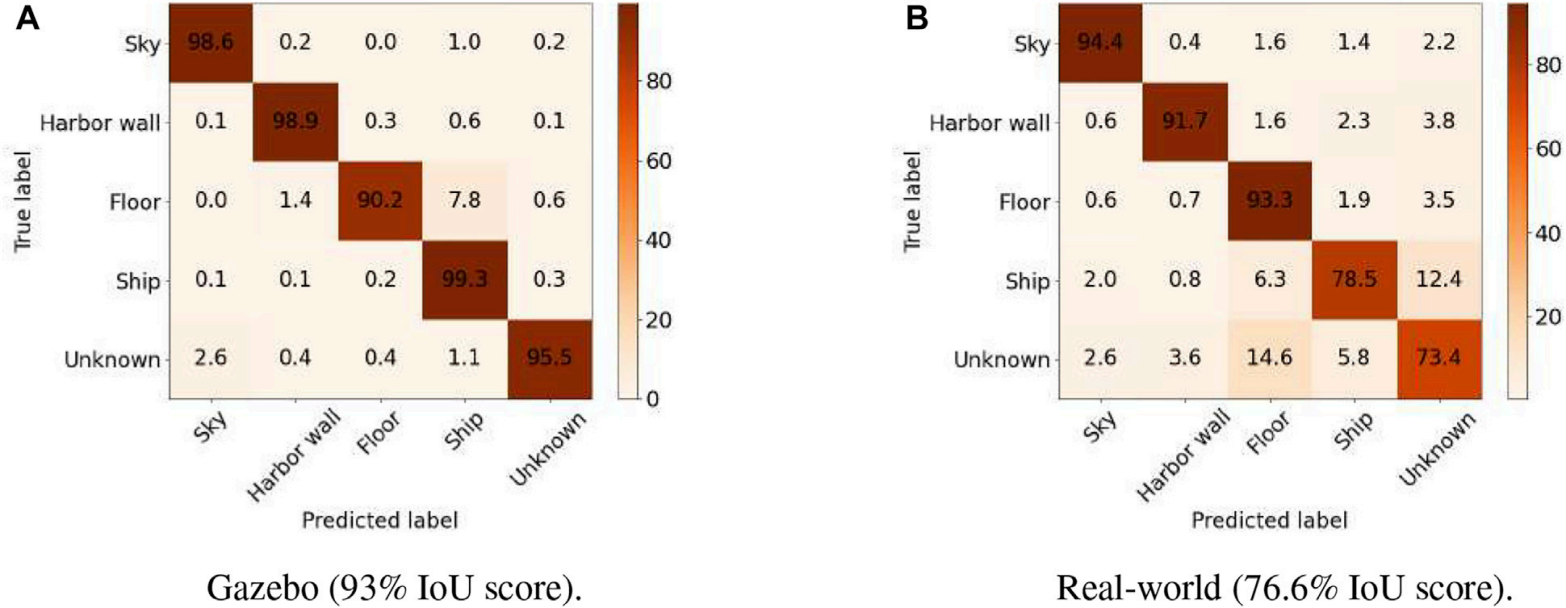
Pixel-level confusion matrix of our semantic network in gazebo and real-world images. **(A)** Gazebo (93% IoU score), **(B)** Real-world (76.6% IoU score).

We quantitatively assessed the performance of our BiseNet network model Yu et al. (2018)) for an input size of 512 × 288. We show the performance on Table 1. In order to assess the performance of our network in semantic segmentation of vessel environments, we split the dataset into two different cases. In the first case (see Table 2), we used the AirSim dataset partition for training, and the Gazebo one for validation and testing, with the end goal of assessing the overall pipeline performance. In the second case (see Table 3), we trained the network with both AirSim (99.9%) and real-world labeled images (0.1%), with the ultimate goal of being able to test the pipeline in a real shipyard environment. We used less than 40% of the real images for training due to the reduced size of the real labeled images dataset. We hypothesize that performance in the real case would significantly improve by increasing the real images dataset size. Figure 4 shows the resulting confusion matrices for both the Gazebo and Real environment test datasets. In the first case, the network is able to successfully learn how to bridge the domain gap between both simulation and real environments, with high accuracy both in simulation (93%) and real domains (76.6%). Furthermore, with only 57 (≈0.001%) real-world labeled training samples, we are able to bridge the gap between AirSim and the real-world environment, achieving an overall accuracy of 76.6 (see Figure 4B). We hypothesize that accuracy would vastly improve by using more real-world training samples and/or domain randomization and adaptation techniques Dehban et al. (2019). We note that the more robust classification performance on the Sky, Floor, and Harbor wall is due to the fact that color and shape properties have significantly less variability and are known *a priori* when compared to the ship and the “pivot” unknown classes. To improve this performance gap, one would need to extend the training dataset for this classes, by either randomizing color and/or extending the shape portfolio in simulation, or by increasing the variability of the real dataset.

**TABLE 1:** Semantic segmentation network performance on the validation (AirSim) and test set (Gazebo).

	Overall Acc	Mean Acc	FreqW Acc	Mean IoU
Val	0.944	0.959	0.896	0.922
Test	0.977	0.965	0.955	0.930

**TABLE 2:** Dataset used for training and validating the semantic segmentation networks.

	Total of images in dataset			Total of images containing category				
	Total	Real	Simulation	Sky	Floor	Ship	Harbor wall	Unknown
Train	58761	57	58704	42495	55066	42349	34048	41961
Val	131	131	0	121	128	102	126	130

**TABLE 3:** Dataset used for training (AirSim), validating (AirSim), and testing (Gazebo) our scene semantic segmentation network for shipyard environments. The dataset specifications include the number of images and classes in each partition.

	# of Images	# Sky	# Floor	# Ship	# Harbor wall	# Unknown
Train	49648	35353	45833	35281	28406	34951
Val	9930	7088	9177	7027	5587	6953
Test	184	175	174	153	177	72

In this work we opted to sacrifice accuracy to achieve low memory consumption and fast computation, which were required for deployment in a real system. Therefore we have chosen BiSeNetv2, instead of other better performing ones such as Mask RCNN. However, other similar architectures such as HarDNet Hong et al. (2021) and Chao et al. (2019), would be suitable alternatives, as demonstrated in le Fevre Sejersen et al. (2021). We emphasize that the main focus of this work was on using probabilistic semantic information for NBV planning.

### 4.2 Multi-camera system evaluation

**FIGURE 5:**
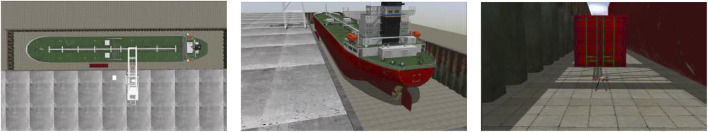
Scenario used for evaluation in (Gazebo/ROS) simulation.

In order to be able to quantitatively and qualitatively measure the performance of the proposed mapping and planning approaches, a realistic shipyard environment (see Figure 5) was created using the Gazebo simulator Koenig and Howard (2004). The environment consists of a dry-dock measuring 145 × 30, ×, 8 m, containing a crane, 1 container, and a dry dock. An intelligent, active mapping algorithm should maximize task-related rewards, in this case, information gathering, by focusing on rewarding viewing directions. We constrain the UAV planner to output positions within this volume since mapping and inspecting the top of the UAV is time-consuming and costly from an economical perspective. More specifically, time should not be spent on mapping and inspecting parts of the ship that can be mapped and repaired outside the dry dock since every hour in the dry dock is extremely costly.

In our experiments, the occupancy probability threshold was set to *P*_*occ*_ = 0.7 and the axial noise standard deviation scaling factor was set to *λ*_*a*_ = 0.005. In each experiment we let the observer collect *T* = 2000 observations (i.e.sense, plan and act iterations). Each experiment was repeated 10 times to average out variability in different simulations due to the randomized nature of our algorithm and non-repeatability influenced by multiple simulation factors, including separate threads for Gazebo’s physics and sensor generation, as well as non-deterministic latencies involved in inter-process communication.

#### 4.2.1 Performance evaluation metrics

We assessed our NBV planning for active and autonomous exploration performance evaluation, using the following metrics:

• the temporal information gain (or temporal entropy reduction):

$$I_{g}=-\underset{m_{i}\in m}{\sum }H_{t}\left(m_{i}\right)$$ (20)

which is a performance metric of the knowledge regarding the surrounding environment, gathered in the probabilistic volumetric map *m*, up to time *t*.

$$I_{g}/T=\frac{1}{T}\overset{T}{\underset{t=1}{\sum }}I_{g_{t}}$$ (21)

When normalized by the number of planning steps it represents the temporal average global information gain per step (i.e., motion planning, sensor acquisition and insertion into the occupancy grid):

• amount of occupied cells (surface coverage)

$$S_{C_{t}}=\underset{m_{i}\in m}{\sum }1\left(P_{t}\left(m_{i}\right)\right)$$ (22)

which is a measure of task-completeness and quantifies the surface covered during reconstruction, where *P*_occ_ represents a user specified probability threshold of the volume being occupied.

$$S_{C}/T=\frac{1}{T}\overset{T}{\underset{t=1}{\sum }}I_{g_{t}}$$ (23)

when normalized by the number of reconstruction steps it represents the average surface coverage per step.

Finally, we evaluate the computational performance (i.e., efficiency) of the methodologies by measuring sensor fusion and planning times.

#### 4.2.2 Receding horizon multi-camera geometric and semantic NBV planning

We first analyzed the influence of different camera setups influence in the trade-off between reconstruction accuracy, planning, and run-time performance. For the number of cameras, we considered *M* ∈ {1; 3; 5}, placed on the front (1), sides (3), back, and bottom (5).

The map resolution was set to *δ* = 0.4*m* to cope with the task requirements. The assessed map information was considered within the bounds of the motion and planning workspace. We compare the performance of our method to the state-of-the-art NBV planning approach of Bircher et al. (2016).

**FIGURE 6:**
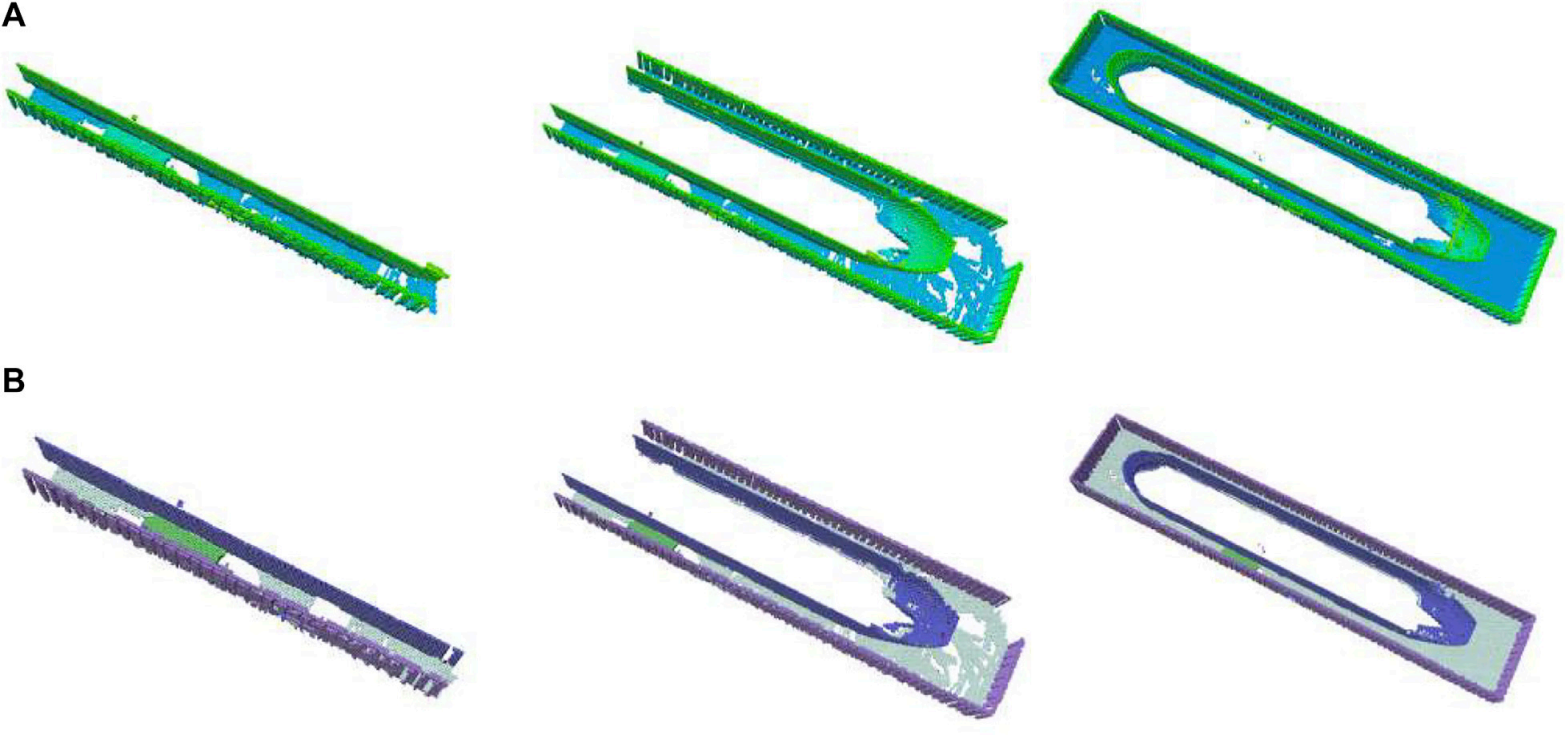
Example reconstruction evolution over NBV planning steps with our method. Octomap colored according to most height **(A)** and most likely semantics **(B)**.

**FIGURE 7:**
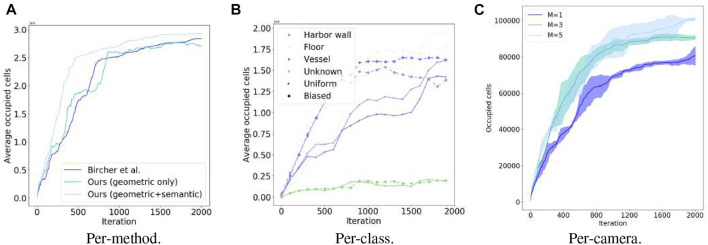
Mapping occupancy performance temporal evolution of our NBV planning in simulation. Each iteration includes planning, act, and sensing acquisition and fusion in the volumetric-semantic grid. **(A)** Per-method, **(B)** Per-class, **(C)** Per-camera.

Figure 6 shows a mapping process until full reconstruction. Figure 7 demonstrates the advantages of utilizing multiple cameras placed around the UAV. For this particular scenario, on average, the use of multiple cameras not only improves coverage quality but also time to full coverage. Full environment mapping is achieved with around 1,600 iterations. As can be seen in Table 4 our semantically informed planning method gathers more information per iteration step, than the semantically agnostic method of Bircher et al. (2016). Also, in qualitative terms, the use of cleverly placed multiple cameras, reduce the number of holes in the map and increases the surface area before full battery depletion.

**TABLE 4:** Average information gain per iteration step.

	*M* = 1	*M* = 3	*M* = 5
Bircher et al. (2016)	15.2	34.5	72.19
Ours (geometric only)	23.9	39.5	82.2
Ours (geometric + semantics)	16.6	45.8	81.08

For evaluating the advantages of incorporating semantics we considered two distributions for the class weights

$$C=\left\{\text{Sky, Floor, Vessel, Harbor Wall, Unknown}\right\}$$ (24)

An unbiased uniform one

$$w_{k}=\left\{0.25,0.25,0.25,0.25,0.25\right\}$$ (25)

to impose a purely geometric-driven exploration task, uninformed to semantics, and a biased one to bias exploration towards structures belonging to vessels

$$w_{k}=\left\{0.1,0.1,0.6,0.1,0.1\right\}$$ (26)

As seen in Table 5 biasing exploration towards specific object classes improves task performance in terms of the mapping accuracy for the class of interest (i.e., vessel). Figure 7B shows resulting reconstructions in the simulated shipyard scenario.

**TABLE 5:** Average mapping computational times (ms) per iteration step.

	Sky	Floor	Ship	Harbor wall	Unknown	Total
Bircher et al. (2016)	-	-	-	-	-	52.3
Ours (geometric only)	-	-	-	-	-	55.6
Ours (geometric + semantic) (Uniform)	0	20.2	16	10	4	53.2
Ours (geometric + semantic) (Vessel Bias)	0	10.7	35.1	4.1	5	57.4

Tables 6 and 7 compares the computational performance of the different mapping approaches for a different number of sensors and observation models. The results qualitatively show that our observational model can obtain good mapping performance compared to the one of Hornung et al. (2013).

**TABLE 6:** Average occupancy per iteration step.

	*M* = 1	*M* = 3	*M* = 5
Baseline Hornung et al. (2013)	43.3	121.5	203.7
Ours (geometric only)	80.2	170.3	290.9
Ours (geometric + semantics)	170.2	230.3	364.9

**TABLE 7:** Average computational times (ms) per iteration step.

	Planning	Mapping
Bircher et al. (2016)	70.3	43.3
Ours (geometric only)	82.2	41.2
Ours (geometric + semantics)	101.5	170.2

### 4.3 Multi-camera drone system design

**FIGURE 8:**
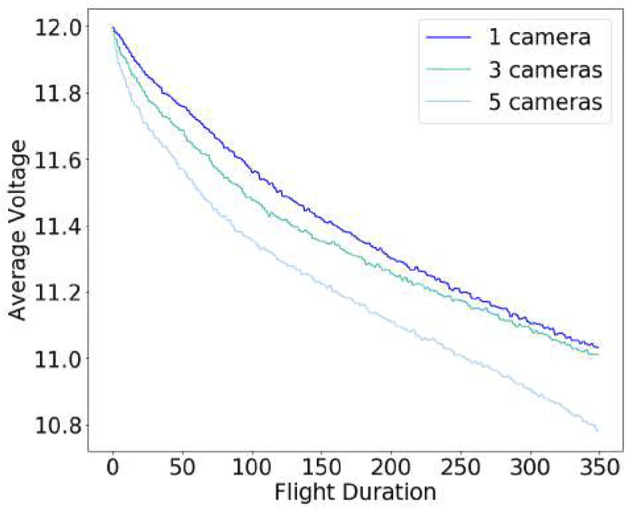
Battery consumption profile.

**FIGURE 9:**
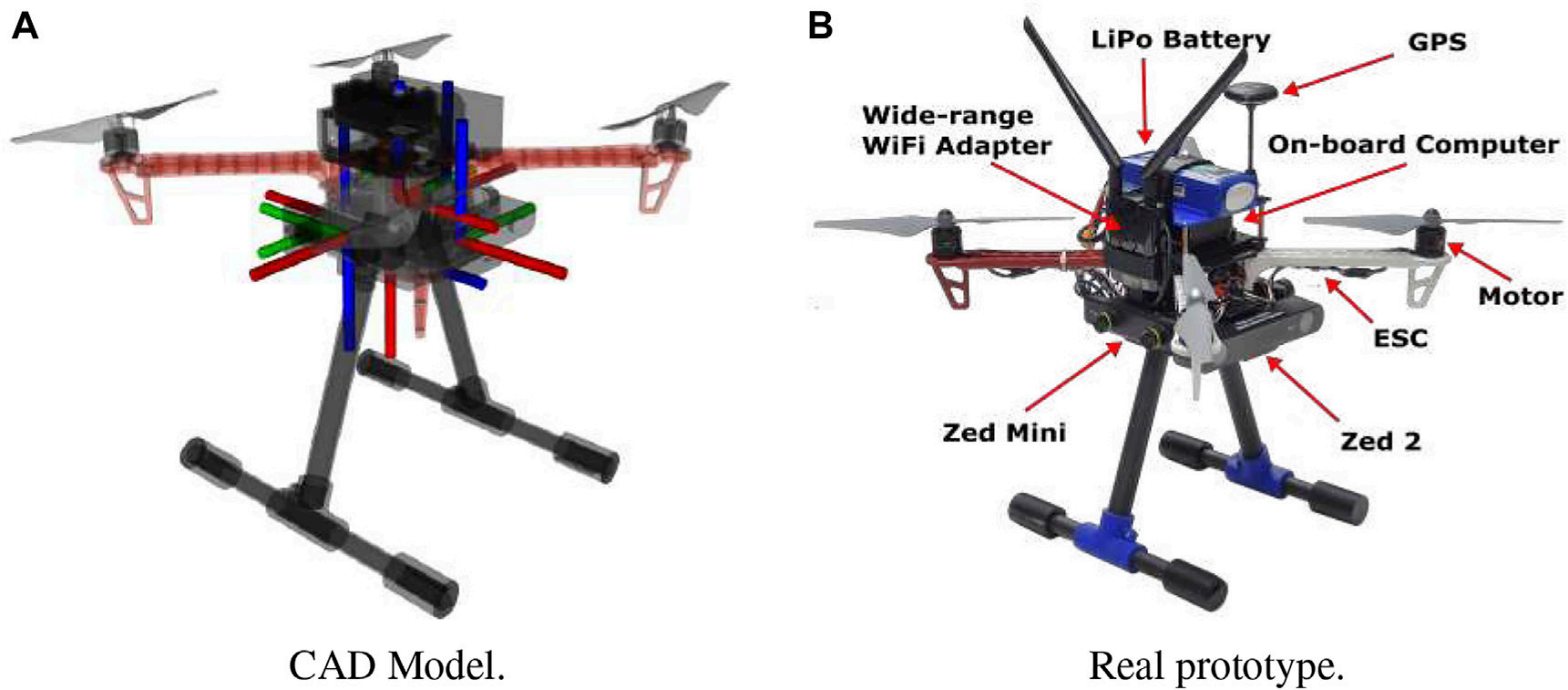
The proposed final drone system design. **(A)** CAD Model, **(B)** Real prototype.

In order to select the most suitable camera configuration for our autonomous flying system, we considered not only time-to-full-coverage results in the previous section but also measured battery power consumption across multiple designs (i.e., different number of sensors) while hovering the UAV with different numbers of cameras (for 5 different runs). As can be seen in Figure 8, the power consumption (proportional to lithium batteries voltage) increases with the number of cameras. Higher total weight and computational processing (including the full localization and mapping system) lead to faster battery depletion. Hence, although improved visibility and faster full environment mapping coverage can be achieved with more cameras (*M* = 5) when considering power constraints and flight duration—which we show in Table 8—*M* = 3 is a more appropriate design choice for this use-case. The final drone design is shown in Figure 9.

**TABLE 8:** Hovering time until battery depletion (minutes).

Cameras	*M* = 1	*M* = 3	*M* = 5
Flight Time	8.79 ± 0.86	8.17 ± 0.41	6.00 ± 0.99

### 4.4 Experiments with real-data

**FIGURE 10:**
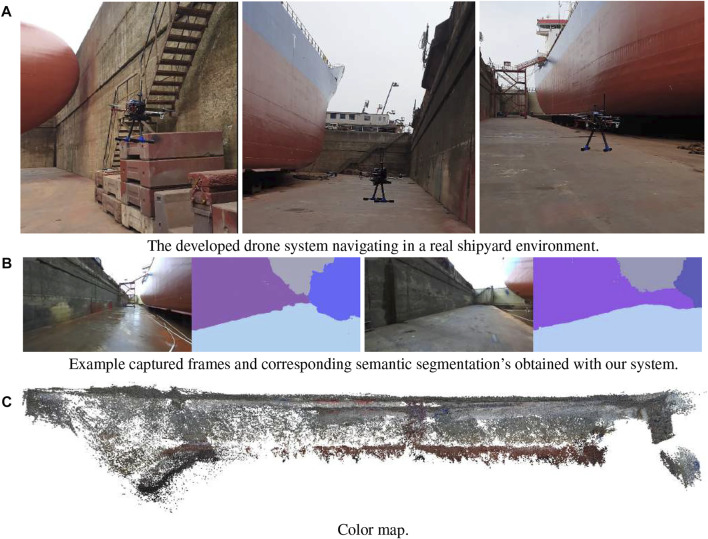
Mapping task performed with our final system design. **(A)** The developed drone system navigating in a real shipyard environment, **(B)** Example captured frames and corresponding semantic segmentation’s obtained with our system, **(C)** Color map.

To validate our system design, we conducted a final experiment in a real dry dock scenario (see Figure 10) located in Fayard, Denmark. The selected multi-camera system design comprised 3 cameras located on the front (Zed 2), left and right (Zed minis) sides of the drone. We let the UAV explore the environment until battery depletion. As can be seen in Figure 10 we were able to successfully build a geometric color map of the dry dock environment, as well as extracting semantics in an 8 min flight. However, due to the impossibility of extracting ground truth data, we were not able to assess the performance of our method in terms of reconstruction accuracy.

## 5 Conclusions and discussion

In this work, we proposed a complete solution for autonomous navigation using multi-camera UAVs that incorporates probabilistic semantic-metric mapping representations for receding horizon NBV planning. The navigation algorithm leverages both semantic and metric probabilistic gains in order to decide where to move the UAV in order to optimize the visual data collection quality of vessel structures in shipyard environments.

We proposed a probabilistic observation model for depth information that allows fast computation of probabilistic range measurements and continuous Bayesian fusion on probabilistic volumetric grid mapping structures. Finally, we introduced a real-time, receding-horizon probabilistic path planning approach that considers both geometric and semantic probabilistic information for planning, using RRTs. Our method is flexible and allows biasing exploration towards specifically known object classes. We assessed the proposed methodologies on a realistic simulation environment (Gazebo) in a set of experiments and demonstrated the benefits of the proposed pipeline for UAV-based inspection tasks, in particular the trade-offs of using single or multiple stereo cameras. To our knowledge, this study is of the utmost importance for resource-constrained unmanned applications, and this work evaluates the previous problem trade-offs on a UAV application scenario. For future work, we intend to explore sensor scheduling acquisition strategies, to decrease computational load. Also, we intend to extend the semantically informed approach to multi-UAV, and try the proposed methodologies in different scenarios.

## Data Availability

Data supporting this article is openly available from the King’s College London research data repository, KORDS, at https://doi.org/10.18742/20448834.

## References

[B1] Aloimonos J. Weiss I. Bandyopadhyay A. (1988). Active vision. Int. J. Comput. Vis. 1, 333–356. 10.1007/bf00133571

[B2] Ashour R. Taha T. Dias J. M. M. Seneviratne L. Almoosa N. (2020). Exploration for object mapping guided by environmental semantics using uavs. Remote Sens. 12, 891. 10.3390/rs12050891

[B3] Bircher A. Kamel M. Alexis K. Oleynikova H. Siegwart R. (2018). Receding horizon path planning for 3d exploration and surface inspection. Auton. Robots 42, 291–306. 10.1007/s10514-016-9610-0

[B4] Bircher A. Kamel M. Alexis K. Oleynikova H. Siegwart R. (2016). “Receding horizon” next-best-view” planner for 3d exploration,” in Robotics and Automation (ICRA), 2016 IEEE International Conference on (Stockholm, Sweden: IEEE), 1462–1468.

[B5] Bloesch M. Omari S. Hutter M. Siegwart R. (2015). “Robust visual inertial odometry using a direct ekf-based approach,” in 2015 IEEE/RSJ International Conference on Intelligent Robots and Systems (IROS), 298–304. 10.1109/IROS.2015.7353389

[B6] Bozcan I. Kayacan E. (2020). “Au-Air: A multi-modal unmanned aerial vehicle dataset for low altitude traffic surveillance,” in 2020 IEEE International Conference on Robotics and Automation (ICRA) (IEEE), 8504–8510.

[B7] Brandao M. Ferreira R. Hashimoto K. Santos-Victor J. Takanishi A. (2013). “Active gaze strategy for reducing map uncertainty along a path,” in 3rd IFToMM International Symposium on Robotics and Mechatronics, 455–466.

[B8] Brandão M. Figueiredo R. Takagi K. Bernardino A. Hashimoto K. Takanishi A. (2020). Placing and scheduling many depth sensors for wide coverage and efficient mapping in versatile legged robots. Int. J. Robotics Res. 39, 431–460. 10.1177/0278364919891776

[B9] Chamoso P. Pérez A. Rodríguez S. Corchado J. M. Sempere M. Rizo R. (2014). “Modeling oil-spill detection with multirotor systems based on multi-agent systems,” in 17th International Conference on Information Fusion (FUSION) (Salamanca, Spain: IEEE), 1–8.

[B10] Chao P. Kao C.-Y. Ruan Y.-S. Huang C.-H. Lin Y.-L. (2019). “Hardnet: A low memory traffic network,” in Proceedings of the IEEE/CVF International Conference on Computer Vision (ICCV).

[B11] Charles R. Q. Su H. Kaichun M. Guibas L. J. (2017). “Pointnet: Deep learning on point sets for 3d classification and segmentation,” in 2017 IEEE Conference on Computer Vision and Pattern Recognition (CVPR), 77–85. 10.1109/CVPR.2017.16

[B12] Chen S. Li Y. Kwok N. M. (2011). Active vision in robotic systems: A survey of recent developments. Int. J. Rob. Res. 30, 1343–1377. 10.1177/0278364911410755

[B13] Dehban A. Borrego J. Figueiredo R. Moreno P. Bernardino A. Santos-Victor J. (2019). “The impact of domain randomization on object detection: A case study on parametric shapes and synthetic textures,” in 2019 IEEE/RSJ International Conference on Intelligent Robots and Systems (IROS), 2593–2600. 10.1109/IROS40897.2019.8968139

[B14] Delmerico J. Isler S. Sabzevari R. Scaramuzza D. (2018). A comparison of volumetric information gain metrics for active 3d object reconstruction. Auton. Robots 42, 197–208. 10.1007/s10514-017-9634-0

[B15] Dornhege C. Kleiner A. (2013). A frontier-void-based approach for autonomous exploration in 3d. Adv. Robot. 27, 459–468. 10.1080/01691864.2013.763720

[B16] Gestwicki P. (2019). Unreal engine 4 for computer scientists. J. Comput. Sci. Coll. 35, 109–110.

[B17] Gutmann J.-S. Fukuchi M. Fujita M. (2005). “A floor and obstacle height map for 3d navigation of a humanoid robot,” in Robotics and Automation, 2005. ICRA 2005. Proceedings of the 2005 IEEE International Conference on. Barcelona, Spain: IEEE, 1066–1071.

[B18] He K. Gkioxari G. Dollar P. Girshick R. (2017). “Mask r-cnn,” in Proceedings of the IEEE International Conference on Computer Vision (ICCV).

[B19] He K. Zhang X. Ren S. Sun J. (2016). “Deep residual learning for image recognition,” in 2016 IEEE Conference on Computer Vision and Pattern Recognition (CVPR), 770–778. 10.1109/CVPR.2016.90

[B20] Herbert M. Caillas C. Krotkov E. Kweon I.-S. Kanade T. (1989). “Terrain mapping for a roving planetary explorer,” in Robotics and Automation, 1989. Proceedings., 1989 IEEE International Conference on (Scottsdale, AZ, USA: IEEE), 997–1002.

[B21] Hong Y. Pan H. Sun W. Jia Y. (2021). Deep dual-resolution networks for real-time and accurate semantic segmentation of road scenes. arXiv Prepr. arXiv:2101.06085.

[B22] Hornung A. Wurm K. M. Bennewitz M. Stachniss C. Burgard W. (2013). Octomap: An efficient probabilistic 3d mapping framework based on octrees. Auton. Robots 34, 189–206. 10.1007/s10514-012-9321-0

[B23] Hou L. Chen X. Lan K. Rasmussen R. Roberts J. (2019). Volumetric next best view by 3d occupancy mapping using Markov chain gibbs sampler for precise manufacturing. IEEE Access 7, 121949–121960. 10.1109/ACCESS.2019.2935547

[B24] Hu J. Li L. Lin Y. Wu F. Zhao J. (2019). “A comparison and strategy of semantic segmentation on remote sensing images,” in The International Conference on Natural Computation, Fuzzy Systems and Knowledge Discovery (Berlin, Germany: Springer), 21–29.

[B25] Isler S. Sabzevari R. Delmerico J. Scaramuzza D. (2016). “An information gain formulation for active volumetric 3d reconstruction,” in Robotics and Automation (ICRA), 2016 IEEE International Conference on (Stockholm, Sweden: IEEE), 3477–3484.

[B26] Karaman S. Frazzoli E. (2011). Sampling-based algorithms for optimal motion planning. Int. J. Robotics Res. 30, 846–894. 10.1177/0278364911406761

[B27] Koenig N. Howard A. (2004). “Design and use paradigms for gazebo, an open-source multi-robot simulator,” in 2004 IEEE/RSJ International Conference on Intelligent Robots and Systems (IROS) (IEEE Cat. No.04CH37566). vol. 3, 2149–2154. 10.1109/IROS.2004.1389727

[B28] Kriegel S. Rink C. Bodenmüller T. Suppa M. (2015). Efficient next-best-scan planning for autonomous 3d surface reconstruction of unknown objects. J. Real. Time. Image Process. 10, 611–631. 10.1007/s11554-013-0386-6

[B29] Kuffner J. J. LaValle S. M. (2000). “Rrt-connect: An efficient approach to single-query path planning,” in Proceedings 2000 ICRA. Millennium Conference. IEEE International Conference on Robotics and Automation. Symposia Proceedings (Cat. No. 00CH37065) (San Francisco, CA, USA: IEEE), 995–1001. vol. 2.

[B30] LaValle S. M. (1998). Rapidly-exploring random trees: A new tool for path planning.

[B31] le Fevre Sejersen J. Pimentel De Figueiredo R. Kayacan E. (2021). “Safe vessel navigation visually aided by autonomous unmanned aerial vehicles in congested harbors and waterways,” in 2021 IEEE 17th International Conference on Automation Science and Engineering (CASE), 1901–1907. 10.1109/CASE49439.2021.9551637

[B32] Li Y. Liu C. (2019). Applications of multirotor drone technologies in construction management. Int. J. Constr. Manag. 19, 401–412. 10.1080/15623599.2018.1452101

[B33] Long J. Shelhamer E. Darrell T. (2015). “Fully convolutional networks for semantic segmentation,” in The IEEE Conference on Computer Vision and Pattern Recognition (CVPR). 10.1109/TPAMI.2016.2572683 27244717

[B34] Maturana D. Scherer S. (2015). “Voxnet: A 3d convolutional neural network for real-time object recognition,” in 2015 IEEE/RSJ International Conference on Intelligent Robots and Systems (IROS), 922–928. 10.1109/IROS.2015.7353481

[B35] Meier L. Honegger D. Pollefeys M. (2015). “Px4: A node-based multithreaded open source robotics framework for deeply embedded platforms,” in 2015 IEEE international conference on robotics and automation (ICRA) (Seattle, WA, USA: IEEE), 6235–6240.

[B36] Michel P. Chestnutt J. Kuffner J. Kanade T. (2005). “Vision-guided humanoid footstep planning for dynamic environments,” in Humanoid Robots, 2005 5th IEEE-RAS International Conference on (Tsukuba, Japan: IEEE), 13–18.

[B37] Mogili U. R. Deepak B. (2018). Review on application of drone systems in precision agriculture. Procedia Comput. Sci. 133, 502–509. 10.1016/j.procs.2018.07.063

[B38] Moravec H. Elfes A. (1985). “High resolution maps from wide angle sonar,” in Robotics and Automation. Proceedings. 1985 IEEE International Conference on (St. Louis, MO, USA: IEEE), 116–121. vol. 2.

[B39] Mur-Artal R. Tardós J. D. (2017). Orb-slam2: An open-source slam system for monocular, stereo, and rgb-d cameras. IEEE Trans. Robot. 33, 1255–1262. 10.1109/TRO.2017.2705103

[B40] Nguyen C. V. Izadi S. Lovell D. (2012). “Modeling kinect sensor noise for improved 3d reconstruction and tracking,” in 3D Imaging, Modeling, Processing, Visualization and Transmission (3DIMPVT), 2012 Second International Conference on (Zurich, Switzerland: IEEE), 524–530.

[B41] O’Callaghan S. Ramos F. T. Durrant-Whyte H. (2009). “Contextual occupancy maps using Gaussian processes,” in Robotics and Automation, 2009. ICRA’09. IEEE International Conference on (Kobe, Japan: IEEE), 1054–1060.

[B52] Pimentel de Figueiredo R. Le Fevre Sejersen J. Grimm Hansen J. Brandão M. Kayacan E. (2021). “Real-time volumetric-semantic exploration and mapping: An uncertainty-aware approach,” in IEEE/RSJ International Conference on Intelligent Robots and Systems (IROS), 9064–9070. 10.1109/IROS51168.2021.9635986

[B42] Quigley M. (2009). “Ros: An open-source robot operating system,” in ICRA 2009.

[B43] Ren S. He K. Girshick R. Sun J. (2017). Faster r-cnn: Towards real-time object detection with region proposal networks. IEEE Trans. Pattern Anal. Mach. Intell. 39, 1137–1149. 10.1109/TPAMI.2016.2577031 27295650

[B44] Ronneberger O. Fischer P. Brox T. (2015). “U-net: Convolutional networks for biomedical image segmentation,” in International Conference on Medical image computing and computer-assisted intervention (Berlin, Germany: Springer), 234–241.

[B45] Scott W. R. Roth G. Rivest J.-F. (2003). View planning for automated three-dimensional object reconstruction and inspection. ACM Comput. Surv. 35, 64–96. 10.1145/641865.641868

[B46] Shah S. Dey D. Lovett C. Kapoor A. (2018). “Airsim: High-fidelity visual and physical simulation for autonomous vehicles,” in Field and service robotics (Berlin, Germany: Springer), 621–635.

[B47] Thrun S. Burgard W. Fox D. (2005). Probabilistic robotics.

[B48] Triebel R. Pfaff P. Burgard W. (2006). “Multi-level surface maps for outdoor terrain mapping and loop closing,” in 2006 IEEE/RSJ international conference on intelligent robots and systems (Beijing, China: IEEE), 2276–2282.

[B49] Yamauchi B. (1997). “A frontier-based approach for autonomous exploration,” in Computational Intelligence in Robotics and Automation, 1997. CIRA’97., Proceedings., 1997 IEEE International Symposium on, 146–151. 10.1109/CIRA.1997.613851

[B50] Yu C. Wang J. Peng C. Gao C. Yu G. Sang N. (2018). Bisenet: Bilateral segmentation network for real-time semantic segmentation. Corr. abs/1808.00897.

[B51] Zhirong W. Song S. Khosla A. Fisher Y. Zhang L. Tang X. (2015). “3d shapenets: A deep representation for volumetric shapes,”, 1912–1920. 10.1109/CVPR.2015.7298801

